# Human PARP1 substrates and regulators of its catalytic activity: An updated overview

**DOI:** 10.3389/fphar.2023.1137151

**Published:** 2023-02-23

**Authors:** Tao Zhu, Ju-Yan Zheng, Ling-Ling Huang, Yan-Hong Wang, Di-Fei Yao, Hai-Bin Dai

**Affiliations:** ^1^ Department of Pharmacy, The Second Affiliated Hospital, Zhejiang University School of Medicine, Hangzhou, China; ^2^ Institute of Clinical Pharmacology, Xiangya Hospital, Central South University, Changsha, China

**Keywords:** PARP inhibitors, poly-ADP ribosylation, substrate, DNA damage repair, synthetic lethality

## Abstract

Poly (ADP-ribose) polymerase 1 (PARP1) is a key DNA damage sensor that is recruited to damaged sites after DNA strand breaks to initiate DNA repair. This is achieved by catalyzing attachment of ADP-ribose moieties, which are donated from NAD^+^, on the amino acid residues of itself or other acceptor proteins. PARP inhibitors (PARPi) that inhibit PARP catalytic activity and induce PARP trapping are commonly used for treating *BRCA1/2*-deficient breast and ovarian cancers through synergistic lethality. Unfortunately, resistance to PARPi frequently occurs. In this review, we present the novel substrates and regulators of the PARP1-catalyzed poly (ADP-ribosyl)ation (PARylatison) that have been identified in the last 3 years. The overall aim is the presentation of protein interactions of potential therapeutic intervention for overcoming the resistance to PARPi.

## 1 Introduction

Poly (ADP-ribose) polymerases (PARPs) are a group of enzymes that may regulate cellular processes such as DNA damage response, chromatin remodeling, cell metabolism and transcriptional regulation (Bai and Canto, 2012; Schiewer et al., 2012). Poly (ADP-ribosyl)ation (PARylation) is central to the key functions of PARPs, about 90% of PARylation produced in a cell is catalyzed by PARP1, the founding member of PARP family. PARP1 catalyzes PARylation by covalently attaching the ADP-ribose moieties to the acceptor amino acid residues on target proteins. Although PARP1 was initially identified as being involved in the sensing and repairing of single strand DNA breaks, PARP1-mediated PARylation may lead to the recruitment of different DNA repair proteins to damaged sites. The overall effect is that PARP1 affects multiple DNA repair pathways, including base excision repair (BER), non-homologous end joining (NHEJ), and homologous recombination (HR) (Masson et al., 1998; Schultz et al., 2003; Wang et al., 2006). As a result, PARP1 has been recognized as a desirable target to achieve DNA damage-induced cell death for anticancer therapy, with several generations of PARP1 inhibitors having been developed and approved in clinical use.

PARP1 inhibitors are characterized by their remarkable efficacy in *BRCA1/2*-mutated breast, ovarian, and prostate cancers. Cancer cells with HR deficiency due to *BRCA1/2* gene mutations are viable by virtue of complementary functions of non-HR DNA repair pathways. However, since PARP1 is involved in non-HR repair, HR deficient cancer cells are extremely vulnerable to PARP1 inhibitors. Currently, there are six FDA-approved PARP inhibitors (olaparib, rucaparib, niraparib, talazoparib, fluzoparib, and pamiparib) for anticancer treatment (Dias et al., 2021; Lee, 2021; Markham, 2021), and several other compounds are being tested in clinical trials such as veliparib (NCT01434316). Unfortunately, despite a dramatic initial response to PARP inhibitors, most patients often develop drug resistance, leading to tumor recurrence. Mechanisms of resistance to PARP inhibitors include restoration of HR capacity, stabilization of replication forks, reduced trapping of PARP1, and P-glycoprotein-mediated drug efflux (Kim et al., 2021). Combination therapies have been recognized as an efficient approach to tackle PARP inhibitors resistance. Accumulating evidence shows superior antitumor efficacy of combinational strategies comprising PARP inhibitors and other kinase or immune checkpoint blockers, such as ATR inhibitors that block BRCA1-independent RAD51 recruitment to DSBs and disrupt fork progression (Yazinski et al., 2017; Kim et al., 2020), and anti-PD-(L)1 antibodies which show a synergistic effect with PARPi (Konstantinopoulos et al., 2019; Domchek et al., 2020; Peyraud and Italiano, 2020).

An increasing number of target proteins that can be PARylated by PARP1 have been identified, further complementing our understanding of the biological function of PARP1. Besides proteins, nucleic acids are also found as substrates of PARylation, which have been comprehensively summarized by Groslambert and colleagues (Groslambert et al., 2021) and are not the focus of this review. Moreover, molecules that regulate PARP1 catalytic activity to influence ADP-ribosylation and PARP1 inhibitor efficacy are being discovered. Herein, we review protein substrates of PARylation catalyzed by PARP1 and regulators of PARP1 catalytic activity identified in the last 3 years, aiming to summarize candidate targets that can be exploited in novel combinational therapies to improve the antitumor efficacy of PARP inhibitors.

## 2 Mechanisms of action of PARP inhibitors

**FIGURE 1:**
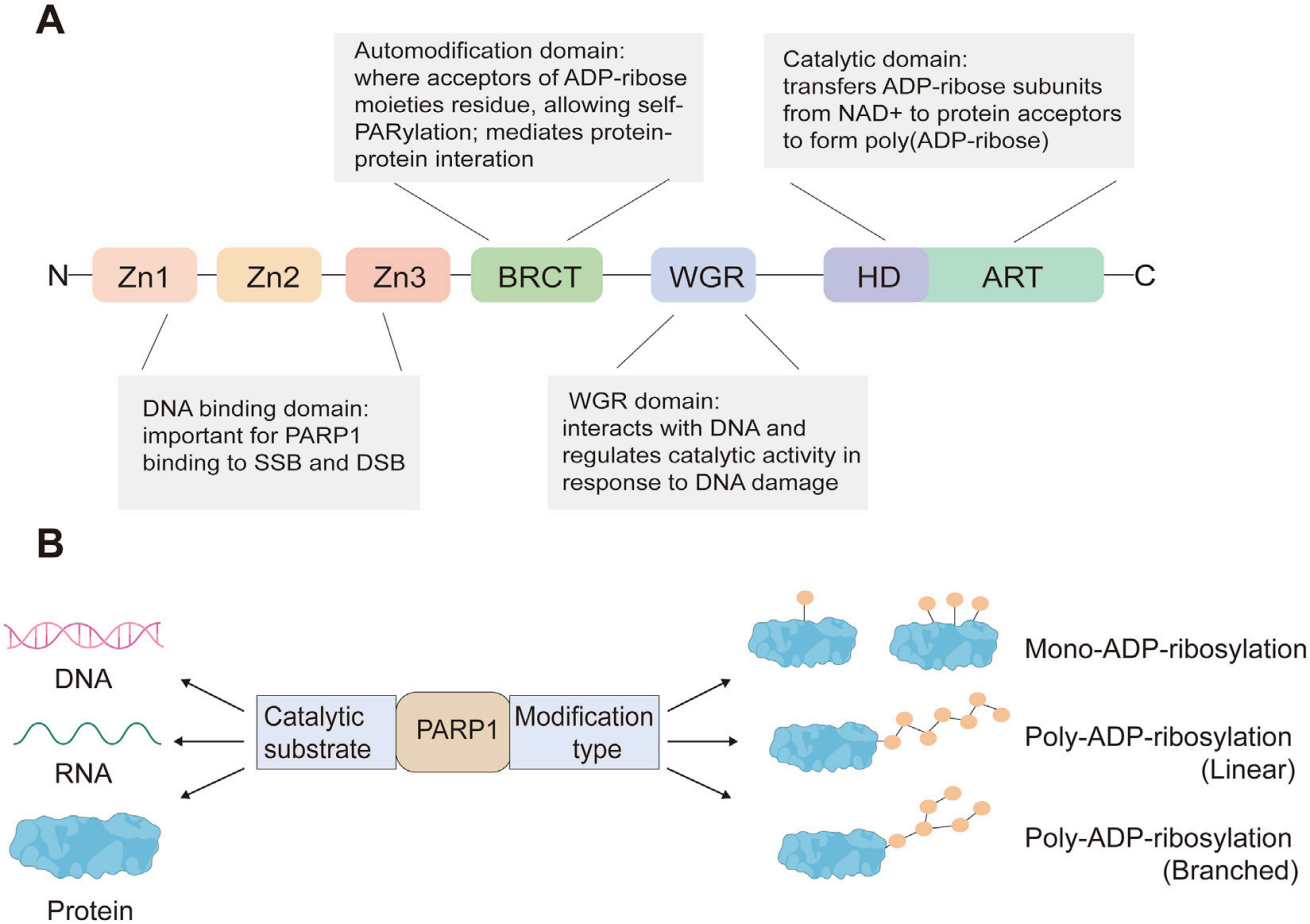
PARP1 protein domains **(A)** and PARP1-mediated ADP-ribosylation modification types of its substrates **(B)**.

**FIGURE 2:**
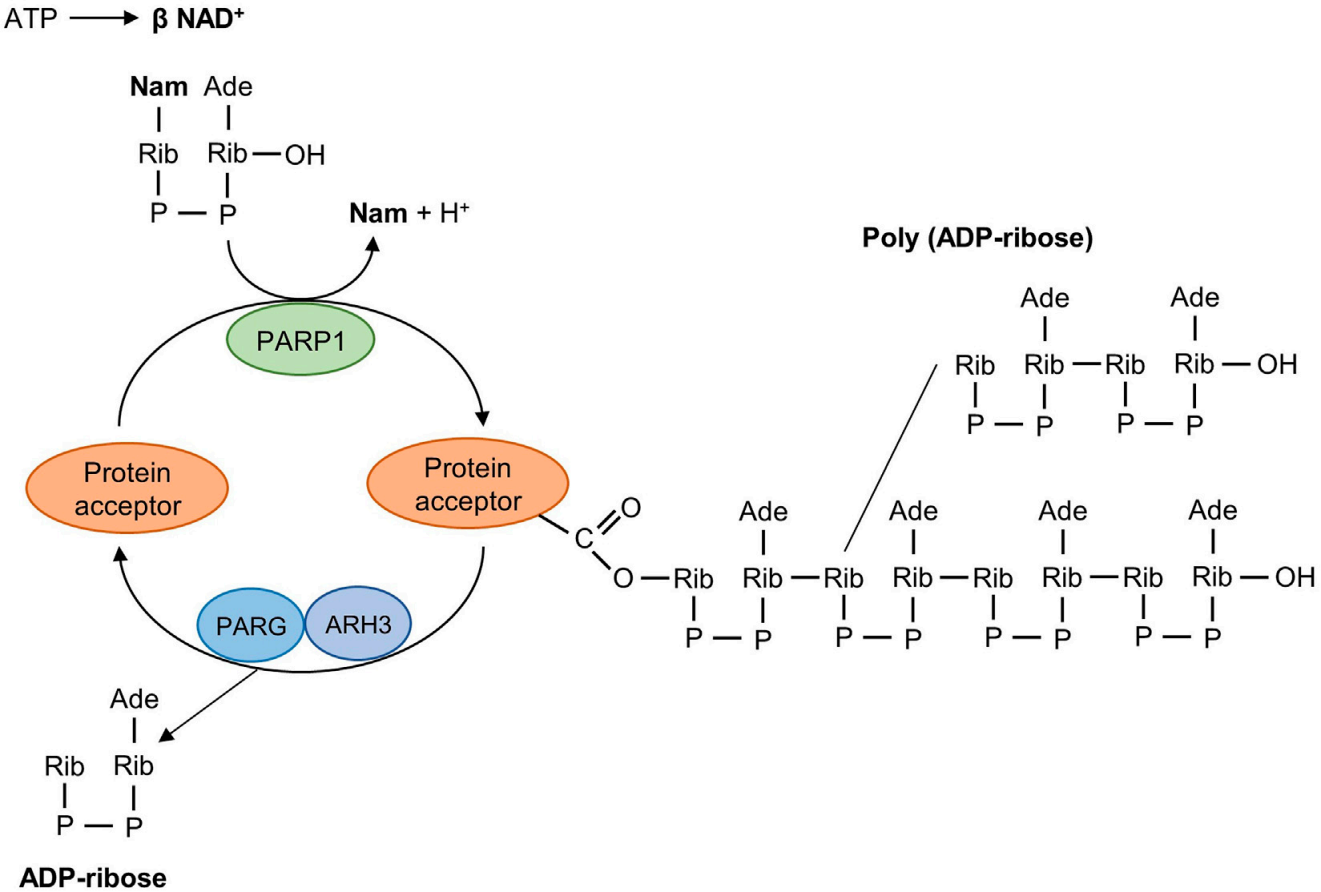
Mechanism of poly (ADP-ribosyl)ation reaction catalyzed by PARP1. PARP1 detects DNA strand breaks, hydrolyses NAD^+^, and catalyzes the transfer of ADP-ribose units on amino acid residues of protein acceptors. The poly (ADP-ribosyl)ation reaction is reversible and the degradative nuclear enzymes PARG and ARH3 cleave poly (ADP-ribose) into ADP-ribose units. PARG, poly (ADP-ribose) giycohydroiase; ARH3, poly (ADP-ribose) hydrolase-3; Nam, nicotinamide; Ade, adenine; Rib, ribose; P, phosphate.

The PARP family consists of 17 enzymes, with a conserved catalytic motif (Schreiber et al., 2006; van Beek et al., 2021) that catalyzes transfer of the ADP-ribose unit from nicotinamide adenine dinucleotide (NAD^+^) onto target proteins. As the first family member, PARP1 is crucial for maintaining genome stability by synthesizing PAR which serves as a docking site for the recruitment of DNA repair effectors to DNA strand breaks. PARP1 has a modular structure with six domains (Figure 1). Three zinc-finger DNA-binding domains (Zn1, Zn2, and Zn3) in the N-terminus are responsible for recognizing particular DNA structures and mediating interdomain contacts (Langelier et al., 2008; Langelier et al., 2011). An adjacent BRCA1 C-terminus (BRCT) domain mediates protein-protein interactions and is the region where PARP1 auto-modification occurs. The Trp-Gly-Arg (WGR) domain also interacts with DNA and regulates the catalytic activity of PARP1 in response to DNA damage (Langelier et al., 2012; Langelier et al., 2018). The C-terminal catalytic domain comprises two subdomains, the auto-inhibitory helical subdomain (HD) and the ADP-ribosyl transferase (ART) subdomain. The conserved ART subdomain bears amino acids that form the catalytic pocket, which interacts with NAD^+^ and catalyzes ADP-ribosylation. HD inhibits the binding of PARP1 with NAD^+^ when PARP1 is in the non-DNA bound state (Ruf et al., 1998; Langelier et al., 2012; Eustermann et al., 2015). In response to DNA strand breaks, PARP1 hydrolyses NAD^+^ and catalyzes covalent attachment of ADP-ribose units on amino acid residues of protein acceptors. This is a dynamic process, the ADP-ribose polymer has a short half-life and is degraded by the poly (ADP-ribose) glycohydrolase (PARG) and the poly (ADP-ribose) hydrolase 3 (ARH3) (Meyer-Ficca et al., 2004; Oka et al., 2006) (Figure 2).

Clinical PARP inhibitors are basically NAD^+^ analogs, all of which contain the nicotinamide moiety (Lord and Ashworth, 2017) (Table 1). PARP inhibitors block the catalytic activity of PARP1 and PARP2 by competitively binding to the NAD^+^-binding catalytic pocket of PARP enzymes, resulting in no formation of PAR polymers and thus no recruitment of DNA damage repair proteins (Min and Im, 2020). PARP inhibitors are lethal to *BRCA*-mutant cancer cells, since they induce single strand DNA lesions, and persistent single strand DNA breaks lead to DSBs that cannot be repaired by impaired homologous recombination. This is so-called synthetic lethality (Farmer et al., 2005). However, later evidence demonstrated that PARP inhibition impeded DNA damage repair and induced cell death to a greater extent than PARP depletion alone (Strom et al., 2011). These data suggest that PARP may exert more activities than its mere enzymatic action.

**TABLE 1:** Chemical structure of the clinical PARP inhibitors.

PARP inhibitor	Structure	References
Olaparib	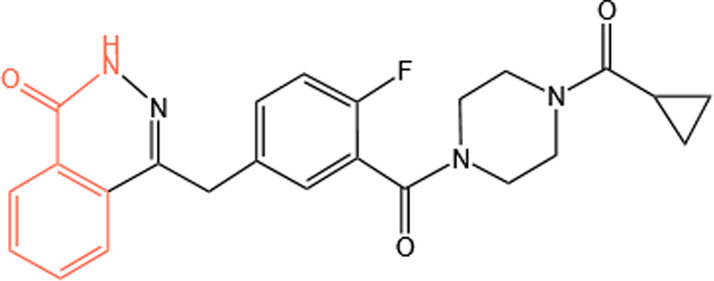	Deeks (2015)
Rucaparib	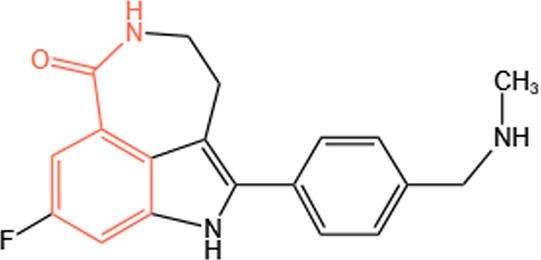	Anscher et al. (2021)
Niraparib	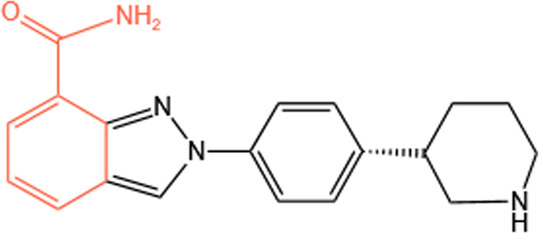	Zhi et al. (2022)
Talazoparib	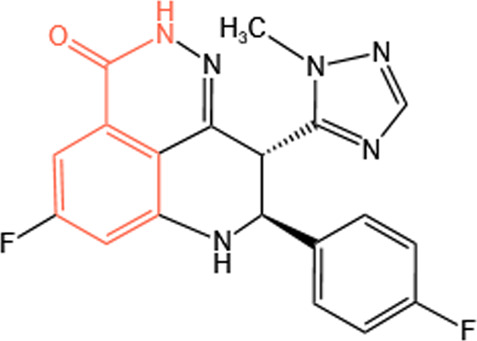	Hoy (2018)
Fluzoparib	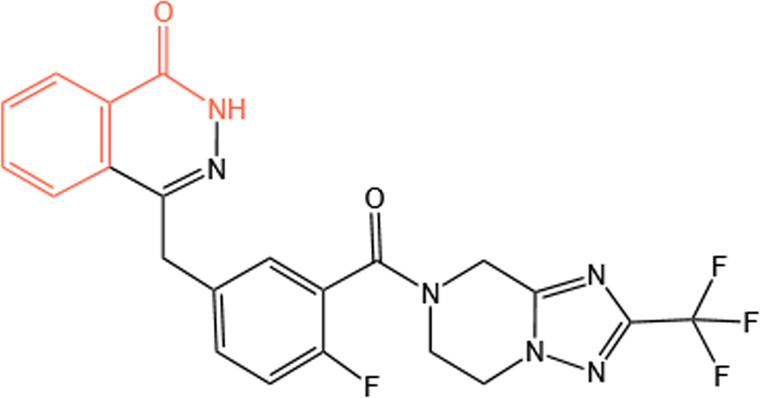	Lee (2021)
Pamiparib	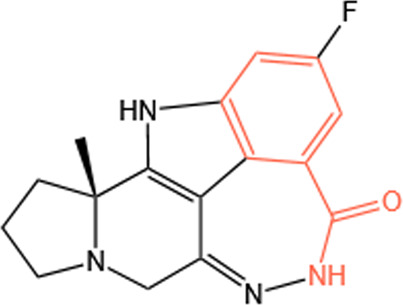	Markham (2021)

The nicotinamide moiety shown in red is common to PARP inhibitors and NAD.

**FIGURE 3:**
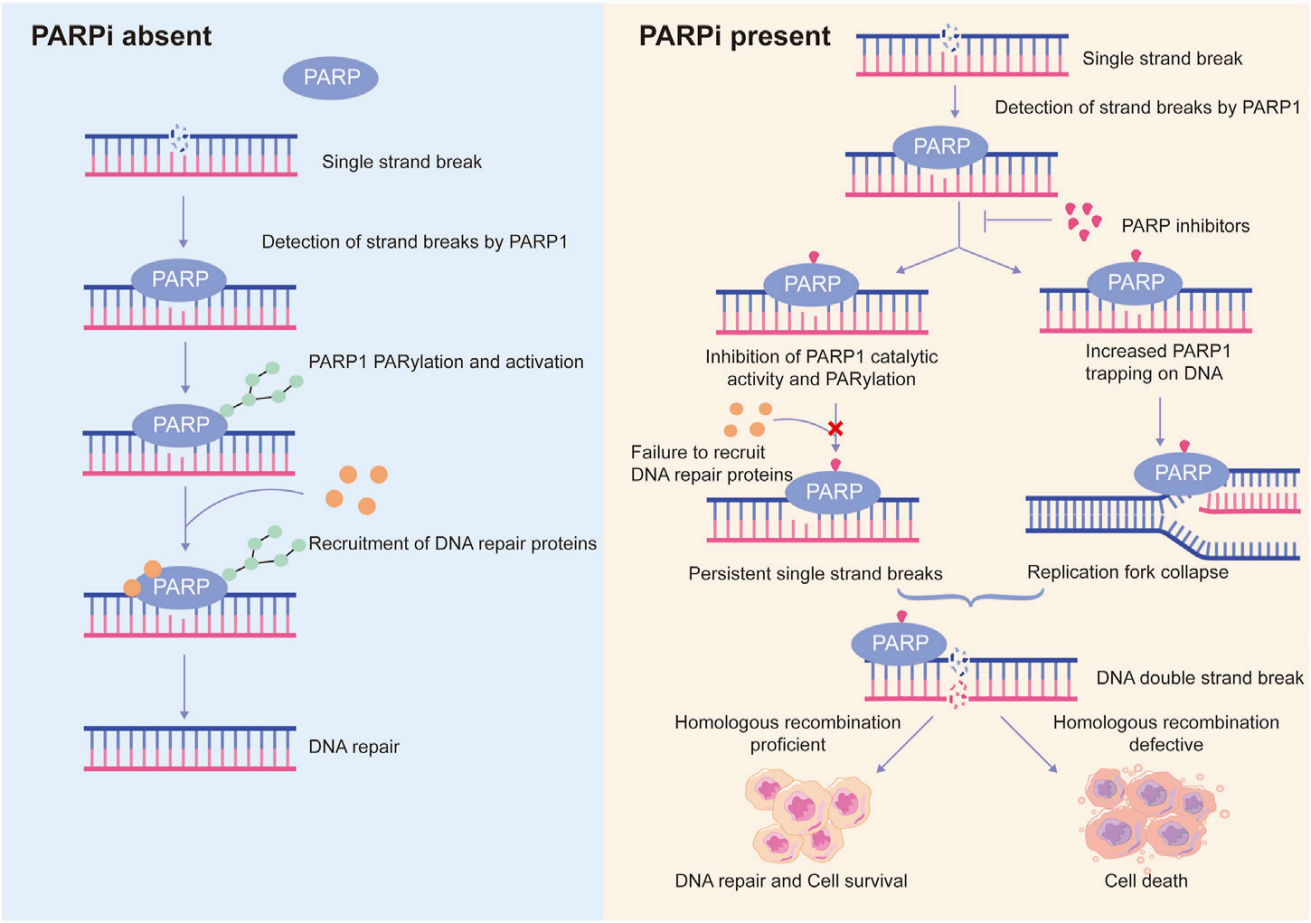
Schematic representation of the proposed mechanisms of action of PARP inhibitors. Normally, PARP1 detects DNA single strand breaks and is activated by them, leading to PARP1 auto-PARylation and recruitment of DNA repair proteins to trigger DNA repair. However, in the presence of PARP inhibitors (PARPi), on the one hand, PARPi suppresses PARP1 activity, recruitment of DNA repair proteins to damaged sites is inhibited, resulting in persistent single strand breaks; on the other hand, PARPi traps PARP1 at DNA lesions, the trapped PARP1-DNA complexes are cytotoxic and cause collapse of replication fork. Persistent single strand breaks and replication fork collapse will ultimately lead to DNA double strand breaks. In homologous recombination-proficient cells, double strand breaks can be efficiently repaired. In contrast, homologous recombination-defective cells are not able to repair double strand breaks efficiently and accurately, leading to cell death. This is a phenomenon known as synthetic lethality.

PARP1-DNA complexes were detected in PARP inhibitors-treated cells and a PARP1-trapping model was hence presented to further explain the synthetic lethality (Helleday, 2011; Murai et al., 2012) (Figure 3). Normally, PARP1 binds damaged DNA and undergoes allosteric switch to activate its catalytic domain, thereby to PARylate and recruit DNA repair proteins such as XRCC1. Subsequent PARP1 autoPARylation leads to its release from DNA due to the repulsion force between highly negatively charged PAR chains, allowing DNA repair and replication to proceed (Eustermann et al., 2015; Lord and Ashworth, 2017). PARP inhibitors trap PARP1 onto DNA, preventing its autoPARylation and release. Although all current PARP inhibitors used in clinical practice are catalytic inhibitors, their ability to trap PARP1 onto DNA varies and is parallel to their cytotoxic potency (Murai et al., 2012). However, it should be noted that PARP1 trapping is linked to catalytic inhibition and is determined by the ability of PARP1 inhibitors to outcompete NAD^+^ binding (Pommier et al., 2016; Xue et al., 2022). Hence, it is rational to reason that molecules regulating PARP1 affinity to NAD^+^ substrate may also affect the PARP1-trapping potency of PARPi, and therefore PARPi cytotoxicity.

## 3 Downstream substrates of PARP1

The protein-targeting domains of PARP1 may constitute the major mechanism by which PARP1 selects specific proteins to modify. PARP1 is targeted to its substrates by the non-catalytic domains, and the regions adjacent to the catalytic domain determine to ADP-ribosylate which amino acids (Cohen and Chang, 2018). Growing evidence has shown the diversity of protein substrates of PARP1, which helps to get insights into the multiple functions of PARP1.

### 3.1 Histones and chromatin remodeling-related proteins

Chromatin is a dynamic DNA scaffold that can modulate the multiple uses of DNA in response to different cellular contexts. Nucleosome is the basic building block of chromatin, which is formed by an octamer of core histones (H3, H4, H2A, and H2B) and 147 bp of DNA that wraps nearly twice around the octamer (Peterson and Laniel, 2004). Posttranslational modification of histones mediates a variety of critical biological processes that are implicated in modifying DNA and regulating gene expression, many of which is dysregulated during cancer progression. For example, a significant correlation of histone modification status with malignant phenotype and clinical outcome was found in breast cancer, with relatively high global histone acetylation and methylation levels associated with a favorable prognosis (Elsheikh et al., 2009). ADP-ribosylation is a less prevalent histone modification, yet all core histones and the linker histone H1 can be ADP-ribosylated (Messner et al., 2010; Li et al., 2018). Histone ADP-ribosylation catalyzed by PARP1 is thought as a way for PARP1 to induce chromatin relaxation and fulfill DNA repair function. H2AX is a histone H2A variant on which posttranslational modifications frequently occur upon DNA damage (Mattiroli et al., 2012; Sone et al., 2014; Ikura et al., 2015). Compared with the H2A nucleosome, PARP1 shows a higher affinity for nucleosomes containing γH2AX, the serine 139 phosphorylation form of H2AX and a sensitive marker of DNA double‐strand breaks (DSBs). This preference renders PARP1 a greater catalytic efficiency (Sharma et al., 2019). Using unbiased high‐resolution mass spectrometry, the glutamate residue 141 (E141) of H2AX has been identified as a novel ADP‐ribosylation site. E141 ADP‐ribosylation facilitates the recruitment of Neil3 glycosylase to the DNA damage sites for removal of damaged base during base excision repair after oxidative DNA damage (Chen et al., 2021). Noteworthily, E141 ADP‐ribosylation and serine 139 phosphorylation of H2AX are mutually exclusive, suggesting that this ADP‐ribosylation also suppresses γH2AX‐involved DSB response (Chen et al., 2021). Histones H3 and H2B are also primary targets that undergo ADP-ribosylation modification on their serine residues in the context of DNA damage. Hananya and colleagues have recently shown that ADP-ribosylation of H2B serine 6 and H3 serine 10 collaboratively restrains chromatin folding and its higher-order organization (Hananya et al., 2021). Their study established that histone mono-ADP-ribosylation is sufficient to inhibit chromatin compaction, and further complemented the previous perspective that PARP1-catalyzed poly-ADP-ribosylation causes chromatin relaxation, which increases the accessibility of repair factors to DNA damage sites (Poirier et al., 1982; Hananya et al., 2021).

PARP1 also mediates ADR-ribosylation of other targets involved in chromatin remodeling. NSD2 is a histone methyltransferase that specifically catalyzes dimethylation of histone H3 lysine 36 (H3K36me2). Its expression plays a role in chromatin accessibility by regulating the balance of H3K36me2 and H3K27me3 modifications (Xie et al., 2022). PARP1 interacts with NSD2 and catalyzes PARylation of NSD2 upon oxidative stress, leading to decreased histone methyltransferase activity of NSD2 and impaired NSD2 recruitment to target genes in multiple myeloma (Huang et al., 2019). This shows an indirect involvement of PARP1 in regulating histone methylation in response to DNA damage.

MORC2 is chromatin remodeling enzyme, with critical roles in gene transcription and DNA damage response through its N-terminal ATPase module (Li et al., 2012; Moissiard et al., 2012). After DNA damage, PARP1 interacts with and recruits MORC2 to DNA damage sites, and PARylates MORC2 at E516 and K517 (Zhang and Li, 2019). PARylation modification stimulates MORC2 ATPase activity to facilitate chromatin remodeling and DNA repair (Zhang and Li, 2019).

In addition, Hu et al. has found that BRD7, a component of the SWI/SNF chromatin remodelling complex, is a substrate of PARylation. PARP1 catalyzes PARylation of BRD7 and enhances its degradation *via* the ubiquitin-proteasome pathway, resulting in resistance to chemotherapy in breast cancer cells (Hu et al., 2019).

ALC1 is a chromatin remodeler recruited to DNA damage sites in a PARylation-dependent manner. PARP1 can PARylate ALC1 to cause the E3 ligase CHFR-mediated ALC1 ubiquitination and degradation (Wang et al., 2019a). In HR-deficient cells, ALC1 is a critical determinant of PARPi cytotoxicity, loss of which reduces cell viability and increases sensitivity to PARPi (Verma et al., 2021).

### 3.2 Transcription factors

Emerging evidence has shown that many transcription factors are substrates of PARP1-mediated PARylation. Signal transducer and activator of transcription (STAT) family of transcription factors is constitutively active in tumorigenesis and promote tumor progression (Heppler and Frank, 2017). In acute myeloid leukemia (AML) with internal tandem duplications of fms-like tyrosine kinase 3 (FLT3-ITD), PARP1 is indispensable for STAT5 activity through interacting and PARylating STAT5 to prevent its proteasomal degradation. Moreover, since PARP1 inhibition constrains STAT5 signaling cascade that contributes to resistance to tyrosine kinase inhibitors (TKIs), it shows a synergistic effect with TKIs for treating AML (Dellomo et al., 2022).

The tumor suppressor p53 guards the genome *via* orchestrating multiple DNA damage repair machineries. It halts cell cycle to allow time for DNA repair and genome stability restoration. P53 can be PARylated by PARP1 in the C-terminal domain, which influences its transcriptional activity (Fischbach et al., 2018). PARylated p53 becomes inactive and induces tumor development in a glioma cell model (Liu et al., 2021).

NFAT5, a transcriptional factor involved in macrophage activation and T-cell development, has been identified as a novel PARylation substrate that mediates PARP1-related DNA damage response. PARP1 PARylates NFAT5 and promotes its recruitment to DNA damage sites where NFAT5 prevents R-loop-associated DNA damage in hepatoma cells (Ye et al., 2021).

In addition, KLF4 is a PARP1-interatcting transcription factor that mediates PARP1 function in controlling telomerase expression (Hsieh et al., 2017). Zhou et al. recently revealed that KLF4 can be PARylated by PARP1 at Y430, Y451, and R452, and KLF4 PARylation is critical for its subcellular location, transcriptional activity, and its function in DNA damage response (Zhou et al., 2020).

HIF-1α is a subunit of the hypoxia-inducible factors (HIF), orchestrating the cell to adapt to hypoxic conditions. It has been shown that PARP1 is a novel regulator in hypoxic adaptation by PARylating HIF-1α at specific K/R residues in the C-terminus domain. This contributes to maintain HIF-1α stability and to enhance its recruitment to target promoters in hypoxia, allowing tumor cells to survive in hypoxic challenges (Marti et al., 2021).

RUNX3 contributes to genome maintenance by regulating the Fanconi anemia (FA) pathway independent of its transcription activity. Multiple PARylable sites have been recognized in RUNX3, and RUNX3 PARylation by PARP1 after DNA damage is crucial for its binding to DNA repair structures and activation of FA pathway-related DNA repair (Zhang et al., 2013; Tay et al., 2018).

ELF4 is a member of the E74-like factor (ELF) transcription factor family that modulates immune cell development and immune responses (Suico et al., 2017). Du et al. found that PARP1 interacts and PARylates ELF4. PARylated ELF4, by transcriptionally regulating elements of DNA damage repair machinery, is pivotal in safeguarding the genome of colon epithelial cells and preventing colitis-associated cancer (Du et al., 2021).

OVOL2 is a negative regulator of mitosis by inhibiting the RHO GTPase signaling (Gugnoni et al., 2022). Multiple PARylable sites within its C2H2 zinc finger domain have been found. PARylated OVOL2 suppresses transcription of SKP2, an E3 ligase of Cyclin E, resulting in centrosome over-duplication and cell death (Zhang et al., 2019).

ER-alpha is an intracellular receptor for hormone estrogen, which promotes cell division and tumor growth through transcriptionally activating its target genes. Recent studies have shown that ER-alpha can be mono- and poly-ADP-ribosylated by PARP1, and its PARylation correlates with tamoxifen resistance (Pulliam et al., 2019; Rasmussen et al., 2021).

### 3.3 Enzymes involved in nucleic acid processing

In addition to nucleic acids such as phosphorylated DNA and RNA ends that serve as substrates of ADP-ribosylation (Groslambert et al., 2021), emerging evidence has shown that ADP-ribosylation can also occur on nucleic acid processing-related enzymes. The DNA polymerase theta (Polθ)-mediated end joining (TMEJ) pathway is essential for DSB repair when the homologous recombination pathway is defective. PARP1 catalytic activity has been shown to facilitate chromosomal TMEJ (Luedeman et al., 2022). However, the helicase domain of Polθ can be PARylated by PARP1, which leads to reduced affinity for single-stranded DNA and impaired ability to bridge DNA overhangs (Schaub et al., 2022). This indicates that PARP1 also negatively regulates TMEJ through Polθ PARylation to maintain appropriate activity of the TMEJ pathway. RNA polymerase III is a binding partner of truncated PARP1, and three subunits (POLR3B, POLR3F, and POLR3G) can be ADP-ribosylated during cytosolic DNA-induced apoptosis (Chen et al., 2022). In addition, PARP1 inhibits elongation of RNA polymerase II *via* suppressing the transcription elongation factor P-TEFb. Upon DNA damage, PARP1 interacts and PARylates the histidine-rich domain of CycT1, a subunit of P-TEFb, disrupting CycT1 phase separation that is required for RNA polymerase II hyperphosphorylation and elongation stimulation (Fu et al., 2022). Interestingly, PARP1 also regulates RNA polymerase elongation independent of its PARylation activity (Matveeva et al., 2022).

DDX21 is a DEAD box-containing RNA helicase that modulates gene transcription and ribosomal RNA processing. PARP1 PARylates DDX21 at its N-terminus, leading to increased PARP1-DDX21 interaction and breast cancer cell proliferation (Kim et al., 2019).

A study by Liu et al. proved that NAT10, an RNA cytidine acetyltransferase, can undergo PARP1-mediated PARylation on K1016, K1017, and K1020 within its C-terminus after DNA damage. NAT10 PARylation enables its nucleoplasmic translocation and increases co-localization and interaction of NAT10 with its substrate MORC2, increasing cell survival in response to irradiation-induced DNA damage (Liu et al., 2022).

### 3.4 Ubiquitination and deubiquitination related enzymes

The ubiquitin (Ub) system regulates protein degradation and signaling pathways to coordinate cellular physiology, which is achieved by a sequential cascade involving Ub-activating enzymes (E1), Ub-conjugating enzymes (E2), Ub ligases (E3), and deubiquitylases (DUBs) (Clague et al., 2015). Accumulating studies indicate a critical role of PARP1 in regulating protein homeostasis through PARylation activity, and increasing enzymes have been identified as substrates of PARylation. E3 ligases NEDD4 and CHFR are such substrates (Correani et al., 2019; Kannan et al., 2019). PARylation of CHFR by PARP1 is important for its activation and mediation of target proteins degradation (Kannan et al., 2019). There is negative feedback between PARP1 and CHFR, as CHFR also mediates PARP1 ubiquitination and degradation (Chung et al., 2021). This is probably because that the PAR-binding pocket of CHFR gives an affinity of CHFR for auto-PARylated PARP1. In other cases, PARP1-mediated PARylation of E3 ligases will prevent their ubiquitylation of target proteins. For example, PARP1 catalyzes PARylation of RNF126 and promotes its proteasome-mediated degradation by recruiting a PAR-binding E3 ligase, resulting in stabilization of targets of RNF126 (Wu et al., 2021). Hence, besides directly PARylating enzymes of the Ub system to participate in the regulation of protein degradation, PARP1-mediated substrate PARylation also serves as bait for recruitment of PAR-binding E3 ligases, such as DTX2 and RNF146 (Hu et al., 2019; Ahmed et al., 2020), to regulate protein turnover.

USP10, a deubiquitylase that can deubiquitinate the tumor suppressor p53, is a PARylation substrate and its stabilization of p53 requires the activity of PARP1 (Correani et al., 2019). BAP1 is a deubiquitylase implicated in DNA repair, in which multiple PARylable sites have been identified. PARP1-mediated PARylation of BAP1 is critical for its deubiquitination activity, protein stability, and recruitment to UV-induced DNA damage sites (Lee et al., 2022). ATXN3 is another DNA repair-related deubiquitylase which dissembles ubiquitin chains on DNA repair substrates. Although no evidence shows that ATXN3 can be PARylated by PARP1, their direct interaction was observed and ATXN3 recruitment to DNA damage sites to mediate retention of DNA repair proteins relies on DNA damage-induced PARylation (Pfeiffer et al., 2021). These above findings suggest a sophisticated role of PARP1 in governing protein degradation either by modulating enzymatic activity in the Ub system or by mediating crosstalk between PARylation and ubiquitination modifications of substrates.

### 3.5 Other substrates

In addition to the substrates mentioned above, several other proteins involved in different cellular processes have been identified as substrates of PARP1-mediated PARylation. These includes players in DNA damage response, such as the DNA-dependent protein kinase DNA-PKcs (Munnur et al., 2019) and the Rho GTPase RAC1 (Marcar et al., 2019). Besides, DNA demethylation enzymes TET1 and TET2 (Tolic et al., 2022), the cytosolic dsDNA sensor cGAS that mediates antiviral immunity (Wang et al., 2022a), and the inflammatory response-involving factor HMGB1 (Kong et al., 2020; Pal Singh et al., 2020) can also be PARylated by PARP1.

**FIGURE 4:**
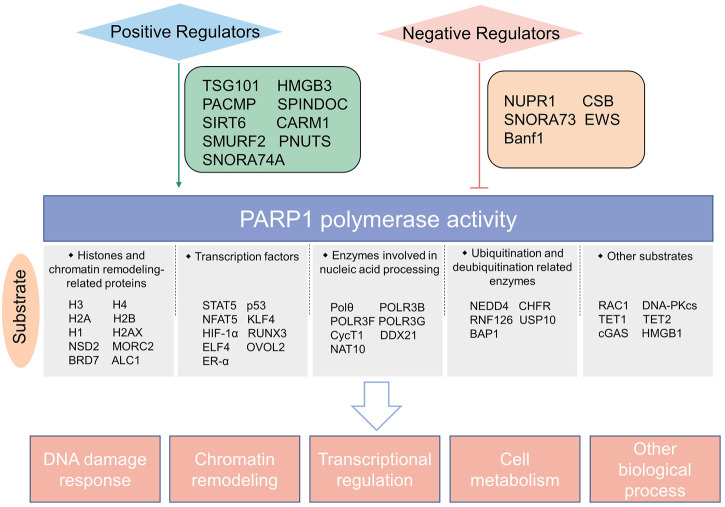
Schematic summary of PARP1 catalytic substrates and regulators of PARP1 enzymatic activity identified in the last 3 years.

With the continuing identification of novel PARylation substrates mediated by PARP1 (Figure 4), a deeper understanding of the roles of PARP1 in DNA damage repair and other biological processes has been achieved, which provides opportunities to develop combination strategies with PARP inhibitors for more effective cancer treatment. However, it should be bear in mind that there is crosstalk between PARP1-mediated PARylation and other posttranslational modifications, which can affect combination therapy efficacy. For instance, PARP1 inhibition suppresses BRD7 PARylation and its ubiquitination degradation, sensitizing BRD7‐positive, rather than BRD7‐negative cancer cells to chemotherapeutic drugs (Hu et al., 2019). This is a case reminding us that cellular contexts should be taken into consideration when exploiting combination strategies.

## 4 Upstream regulators of PARP1 catalytic activity

DNA binding induces conformational changes in the catalytic domain of PARP1 that initiate PARylation of acceptor proteins. Hence, PARP1 catalytic activity is dependent on DNA-binding domains that identify and binds DNA strand breaks. In addition, factors affect the catalytic domain itself and the allosteric signals are also determinants of PARP1 catalytic activity. In this section, we will summarize recently identified upstream regulators of PARP1 activity.

### 4.1 Positive regulators of PARP1 catalytic activity

Increasing studies have revealed the upstream molecules that can enhance the enzymatic activity of PARP1. The tumor susceptibility gene TSG101 interacts with PARP1 and is essential for PARP1 activation. Its loss markedly abolishes cellular PARylation and induces PARP1 trapping in DNA lesions, leading to DNA repair impairment and cell apoptosis (Tufan et al., 2022). HMGB3 is a novel interactor of PARP1 that can stimulate the PARylation activity of PARP1 and inhibit PARP trapping, resulting in olaparib resistance in ovarian cancer (Ma et al., 2022). A recent study by Zhang and colleagues found that PACMP, a lncRNA-derived micropeptide, is an activator that promotes PARP1-meidiated PARylation, and PACMP inhibition renders sensitivity of cancer cells to diverse chemo- and targeted therapies (Zhang et al., 2022). SPINDOC is a component of the histone-code effector protein complex SPIN1. Yang et al. found a SPIN1-independent role for SPINDOC in DNA damage response, which is achieved by directly interacting with PARP1 and facilitating PARP1-mediated PARylation (Yang et al., 2021). Furthermore, the E3 ligase SMURF2, although responsible for ubiquitination and degradation of PARP1 (Qian et al., 2020), can stimulate the enzymatic activity of PARP1 by reducing its monoubiquitination (Ilic et al., 2021). CARM1 is an arginine methyltransferase and functions in regulating DNA replication fork speed through enhancing PARP1 activity, by both enhancing DNA binding of PARP1 and acting corporately with HPF1, a regulator of PARP-1-dependent ADP-ribosylation (Genois et al., 2021). Kong et al. found that the chromatin-associated protein SIRT6 is an upstream signal for PARP1 activation through monoADP-ribosylation (Kong et al., 2020). However, this is challenged by a later study which showed that SIRT6 does not regulate PARP1 activation (Koczor et al., 2021). Hence, further more comprehensive research is needed. Small nucleolar RNAs, such as SNORA74A, were reported to interact with PARP1, serve as activators of PARP-1 catalytic activity, and regulate ribosome biogenesis and cell growth (Kim et al., 2019). In addition, the DNA damage response-involving protein PNUTS is found as a PARP1-binding partner. It is recruited to DNA lesions in a PARP1-dependent fashion and is essential for PARylation modification in response to DNA damage (Wang et al., 2019b), suggesting a possible role of PNUTS in stimulating PARP1 activity. Further endeavors are needed to evaluate if these positive regulators of PARP1 enzymatic activity mentioned above may serve as potential targets to increase anticancer efficacy of PARP inhibitors.

### 4.2 Negative regulators of PARP1 catalytic activity

To avoid hyper-PARylation, cells developed mechanisms to repress PARP1 activity. NUPR1 is a nuclear stress protein which is able to bind to PARP1 and inhibit its enzymatic activity. Pharmacological inhibition of NUPR1 causes deleterious PARylation, mitochondrial dysfunction and cell death (Santofimia-Castano et al., 2022). Han et al. found that SNORA73, a chromatin-associated small nucleolar RNAs, restrains PARP1 auto-PARylation and contributes to genome instability in hematopoietic malignancy (Han et al., 2022). Moreover, the chromatin remodeler CSB was demonstrated as a PARP1-interacting partner. CSB prevents PARP1 overactivation in initial response to oxidative stress, but later CSB helps to maintain chromatin PAR levels (Lake et al., 2022). A study in *Ews* ^−/−^ embryonic tissues by Lee et al. found that EWS suppresses PARP1 activity and reduces DNA damage level by preventing excessive PARP1 accumulation on DNA. Loss of EWS leads to PARP1 hyperactivation and excessive PARylation (Lee et al., 2020). Bolderson et al. reported that Banf1, a DNA-binding protein, interacts directly with the NAD^+^-binding domain of PARP1 and inhibits PARP1 activity, causing defective repair of oxidative DNA lesions (Bolderson et al., 2019).

As described above, PARP1 has multiple interacting partners which can either promote or suppress the activity of PARP1, suggesting that the innate PARP1 catalytic activity is under cunning orchestration (Figure 4). In-depth understanding of this can offer insights to develop strategies to manipulate PARP1 activity more precisely.

## 5 Conclusion and perspectives

PARP inhibitors have shown superior efficacy in patients with breast, ovarian, and prostate cancer, especially those with *BRCA1/2* mutations. However, resistance to PARPi is common, and several mechanisms to explain this phenomenon have been proposed, such as increased drug efflux, loss of PARP1 function, HR reactivation, stabilization of the replication fork, and inactivation of PARG (Chiappa et al., 2021). To overcome PARPi resistance and improve therapeutic efficacy, multiple strategies combining PARPi and other inhibitors have been designed and evaluated under clinical trials (Wang et al., 2022b). In this review, we summarized PARylable substrates of PARP1 and regulators of PARP1 catalytic activity identified in the last 3 years, which we believe will advance the comprehensive understanding of function of PARP1 and offer clues to guide design of pre-clinical and clinical trials to reverse PARPi resistance.

The function of PARP1 other than its DNA repair ability is being revealed with the continuous identification of its enzymatic substrates. We propose that a systematic knowledge of PARP1 function is a prerequisite for us to thoroughly comprehend how PARPi works. According to the PRIMA trial, among newly diagnosed advanced ovarian cancer patients that are responsive to platinum-based chemotherapy, PARPi (niraparib) treatment significantly prolong progression-free survival of patients with or without HR deficiency (Gonzalez-Martin et al., 2019). This suggests a possible cytotoxic effect of PARPi independent of its DNA repair inhibition (Li et al., 2020). Moreover, adverse events caused by PARPi due to on-target effect are a concern that needs attention. We believe that a broad spectrum of substrate proteins of PARP1-catalzyed PARylation may explain the incidence of adverse drug reactions. In future studies, it is necessary to determine whether there are critical substrates with strikingly high contribution weight in mediating PARP1’s DNA repair and other functions that are exploited by PARPi. Compared to PARPi, targeting these substrates may offer an alternative avenue to avoid adverse events without compromising the anti-tumor efficacy.

Regulators of PARP1 catalytic activity are important factors that affect PARPi efficacy, as PARPi cytotoxicity is dependent on cellular PARP1 polymerase activity (Chen et al., 2019). Accumulating positive and negative modulators of PARP1 enzymatic activity have been identified, although most of which are based on *in vitro* cell model or *in vivo* mouse model. If these regulators can be further validated in models more resembling human conditions, it should be better to take them into consideration to achieve the maximal efficacy of PARPi.

In conclusion, we still have a long way to go to cure HR defective cancers with PARPi. However, a deep insight into the downstream PARylable substrates of PARP1 and the critical upstream molecules influencing PARP1 polymerase activity will aid in design of more effective PARPi-based anti-tumor strategies and accelerate our journey to that end.
